# Manipulation of Autophagy in Phagocytes Facilitates Staphylococcus aureus Bloodstream Infection

**DOI:** 10.1128/IAI.00358-15

**Published:** 2015-08-12

**Authors:** Kate M. O'Keeffe, Mieszko M. Wilk, John M. Leech, Alison G. Murphy, Maisem Laabei, Ian R. Monk, Ruth C. Massey, Jodi A. Lindsay, Timothy J. Foster, Joan A. Geoghegan, Rachel M. McLoughlin

**Affiliations:** a Host Pathogen Interactions Group, School of Biochemistry and Immunology, Trinity Biomedical Sciences Institute, Trinity College Dublin, Dublin, Ireland; b Department of Biology and Biochemistry, University of Bath, Claverton Down, Bath, United Kingdom; c Institute for Infection and Immunity, St. George's, University of London, London, United Kingdom; d Microbiology Department, Moyne Institute of Preventative Medicine, Trinity College Dublin, Dublin, Ireland

## Abstract

The capacity for intracellular survival within phagocytes is likely a critical factor facilitating the dissemination of Staphylococcus aureus in the host. To date, the majority of work on S. aureus-phagocyte interactions has focused on neutrophils and, to a lesser extent, macrophages, yet we understand little about the role played by dendritic cells (DCs) in the direct killing of this bacterium. Using bone marrow-derived DCs (BMDCs), we demonstrate for the first time that DCs can effectively kill S. aureus but that certain strains of S. aureus have the capacity to evade DC (and macrophage) killing by manipulation of autophagic pathways. Strains with high levels of Agr activity were capable of causing autophagosome accumulation, were not killed by BMDCs, and subsequently escaped from the phagocyte, exerting significant cytotoxic effects. Conversely, strains that exhibited low levels of Agr activity failed to accumulate autophagosomes and were killed by BMDCs. Inhibition of the autophagic pathway by treatment with 3-methyladenine restored the bactericidal effects of BMDCs. Using an *in vivo* model of systemic infection, we demonstrated that the ability of S. aureus strains to evade phagocytic cell killing and to survive temporarily within phagocytes correlated with persistence in the periphery and that this effect is critically Agr dependent. Taken together, our data suggest that strains of S. aureus exhibiting high levels of Agr activity are capable of blocking autophagic flux, leading to the accumulation of autophagosomes. Within these autophagosomes, the bacteria are protected from phagocytic killing, thus providing an intracellular survival niche within professional phagocytes, which ultimately facilitates dissemination.

## INTRODUCTION

Staphylococcus aureus causes a wide range of pathologies from superficial skin infections to more serious invasive infections associated with significant morbidity and mortality. In severe cases, localized infections can lead to bacterial invasion of the vascular system, causing life-threatening conditions such as bacteremia and sepsis. A key factor facilitating this dissemination is the impressive arsenal of immune evasion strategies available to S. aureus that enables it to evade recognition and killing by the host immune system (1). Identifying and disarming the mechanisms by which this organism circumvents the host's immune system are important strategies for identifying novel therapies.

Although classically considered an extracellular bacterium, S. aureus is capable of invading and persisting within a variety of nonprofessional phagocytic host cells (2), facilitating tissue persistence and relapsing disease. Strikingly, this organism is also capable of manipulating professional phagocytes, and there is evidence that S. aureus can survive within monocytes, macrophages, and even neutrophils (3–6). Unlike resident tissue cells, professional phagocytes are mobile and represent an opportunity for the bacterium to disseminate from the primary focus of infection to systemic sites. In a mechanism similar to that employed by traditional intracellular bacteria such as Mycobacterium tuberculosis and Listeria monocytogenes, which utilize monocytes to disseminate via the bloodstream (7, 8), it has been proposed that S. aureus may be capable of subverting neutrophils to facilitate its dissemination (9). S. aureus has also been shown to persist within human monocyte-derived macrophages (3), suggesting that these cells may also provide a potential intracellular niche to facilitate S. aureus dissemination *in vivo*. The bulk of the research conducted into the survival of S. aureus within or killing of S. aureus by phagocytes has focused on neutrophils and, to a lesser extent, macrophages. To date, the contribution of dendritic cells (DCs) to the direct killing of S. aureus and the capacity of S. aureus to manipulate these particular phagocytes have not been explored.

Despite the fact that the environment inside phagocytes is less than hospitable, gaining an intracellular niche, even briefly, within these cells affords a window of opportunity for extended survival and potential dissemination. Critical to survival is the ability to avoid destruction within phagolysosomes, and S. aureus is equipped with a number of strategies to resist phagolysosomal killing (10–12). Having circumvented these killing mechanisms, the bacterium can then escape into the cytoplasm, which, in most cases, eventually leads to host cell death, releasing the bacteria into the extracellular space, where they have the opportunity to replicate and infect other host cells. Phagosomal escape by S. aureus has been shown to depend upon the regulatory system encoded by the *agr* locus (3, 13, 14), which controls the expression of a number of virulence factors, including the secreted toxin alpha-hemolysin (Hla), a critical effector molecule essential for S. aureus survival within macrophages (3). Phenol-soluble modulins (PSMs) are small cytotoxic alpha-helical peptides. They are categorized into two classes, PSMα and PSMβ peptides. PSMα peptides are regulated by the Agr system and enable phagosomal escape of S. aureus from both nonprofessional (15) and professional (16, 17) phagocytes. Survival within neutrophils appears to be dependent upon the accessory regulator SarA, which facilitates the survival of S. aureus within large vacuoles that are not competent for fusion with lysosomes (5). While it is clear that phagocytes are critically important for effective clearance of S. aureus during an infection, it may be that the intracellular locale of the bacterium postphagocytosis will dictate whether or not the phagocytes contribute to host protection or inadvertently play a deleterious role.

Autophagy is an important homeostatic process in eukaryotic cells that is critical for cell survival. Damaged cytosolic components are removed and recycled in double-membrane vacuoles, called autophagosomes, that are characterized by the recruitment of microtubule-associated protein 1 light chain 3 (LC3) conjugated to phosphatidylethanolamine (LC3-II) to their membrane (18). These autophagosomes then fuse with lysosomes and are digested. This process of autophagosome formation and eventual degradation is termed autophagic flux (19). Autophagy also plays an important role in host defense against bacteria that can invade host cells, such as Streptococcus pyogenes (20), or facultative intracellular pathogens, such as Mycobacterium tuberculosis (21). These organisms are sequestered in autophagosomes, which then deliver the bacteria to the lysosomes for destruction. Some microorganisms (e.g., Coxiella burnetii and Porphyromonas gingivalis) have evolved mechanisms to subvert the autophagic machinery of the cell, delaying autophagosomal maturation and lysosomal fusion and thus creating a survival niche within autophagosomes (22). S. aureus can localize to autophagosomes and inhibit lysosomal fusion within HeLa cells, while proliferation of S. aureus was impaired within fibroblasts deficient in the autophagy protein Atg5 (23), indicating an essential role for the autophagy pathway in facilitating the intracellular survival of S. aureus within nonprofessional phagocytic cells. In this study, a strain that expresses low levels of *agr* failed to colocalize with autophagosomal markers, identifying the requirement for Agr-regulated genes to engage autophagosomes.

Whether or not S. aureus can manipulate the autophagic process in professional phagocytes as a means to evade killing remains to be established. We hypothesized that subversion of autophagy in professional phagocytes could provide S. aureus a means to preserve a temporary intracellular survival niche, in order to facilitate dissemination. We demonstrate the strain-dependent ability of S. aureus to induce the accumulation of autophagosomes in phagocytes, which appears to correlate with interstrain differences in Agr expression. Strains with high levels of Agr activity became associated with autophagosomes, were not killed by phagocytic cells *in vitro*, and demonstrated extended intracellular survival within phagocytes *in vivo*.

## MATERIALS AND METHODS

### Bacterial strains.

S. aureus strains SH1000 (clonal complex 8 [CC8]) and PS80 (CC30) were described previously (24, 25). S. aureus clinical isolates were obtained from blood culture bottles of patients diagnosed with S. aureus bacteremia at St George's Healthcare NHS Trust, London, United Kingdom. Two isolates were used repeatedly throughout this study: Sa68 and Sa279. Both of these strains are methicillin sensitive and belong to the CC1 lineage.

The expression of enhanced green fluorescent protein (GFP) (26) in the PS80 background was achieved through the integration of a nonreplicative integrase vector (pIMC11-GFP) into the phage 11 attachment site. Expression of enhanced GFP is under the control of the P_xyl/tetO_ promoter, without repression from TetR. Chromosomal integration of PS80::pIMC11-GFP was validated with oligonucleotides IM293 and IM294, which amplify across the site of integration, yielding a 0.7-kb product in PS80 and a 3.4-kb product in PS80::pIMC11-GFP.

Deletion of the *agr* locus (*agrBDCA* genes) within PS80 was achieved by allelic exchange using pIMAY (27). Primers agr1 and agr2 amplified 532 bp of DNA upstream of *agrB*, and primers agr3 and agr4 amplified 535 bp of DNA located downstream of the *agrA* gene (Table 1). The PCR products were denatured and allowed to reanneal via the complementary sequences in primers agr2 and agr3. This was used as the template for PCR using primers agr1 and agr4. The amplimer was cloned into pIMAY (27) between the SalI and EcoRI restriction sites by using sequence- and ligase-independent cloning (28), and the resulting plasmid (pIMAY::Δ*agr*) was transformed into DC10B cells and verified by DNA sequencing. The plasmid was transformed into electrocompetent PS80 cells, and deletion of the *agr* genes was achieved by allelic exchange, as previously described (27). The deletion was confirmed by DNA sequencing of a PCR amplimer generated by using PS80Δ*agr* genomic DNA as the template and primers agr OUT F and agr OUT R. The mutant did not produce delta-hemolysin on sheep blood agar.

**TABLE 1:**
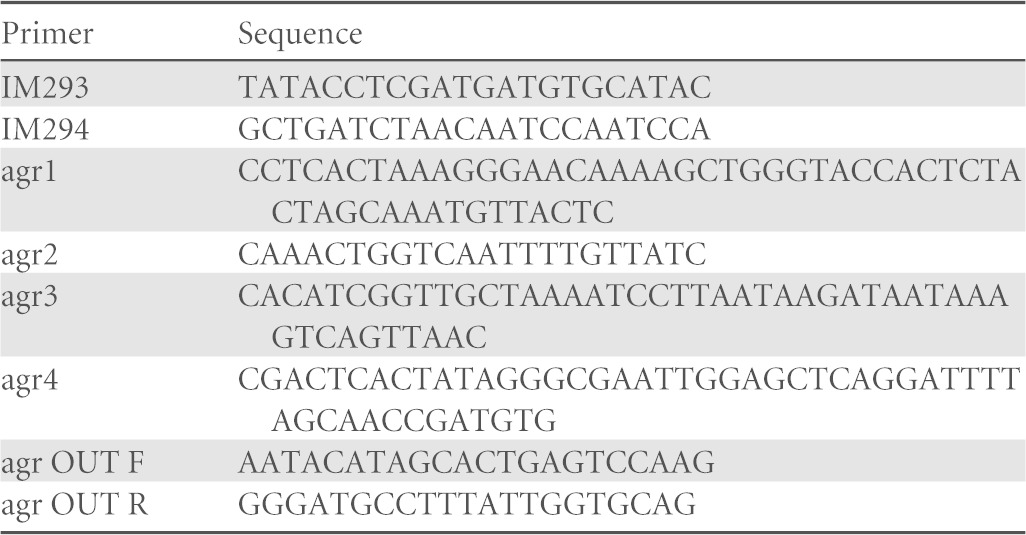
Primers used in mutation of PS80

All bacteria were cultivated from frozen stocks for 24 h at 37°C on agar plates. Bacterial suspensions were then prepared in phosphate-buffered saline (PBS), and the concentrations were estimated by measuring the absorbance of the suspension at 600 nm. CFU were determined by plating serial dilutions of each inoculum.

In the case of PS80-GFP^+^, log-phase growth was required for optimal GFP expression. A single colony was inoculated into tryptic soy broth (TSB) overnight, and a subculture in fresh TSB was taken the following morning. The concentration of bacteria in the broth was determined by measuring the absorbance at 600 nm and confirmed by streaking on agar plates.

For immunofluorescence analysis, bacteria were stained with Cell Trace Violet (CTV; Life Technologies). Stationary-phase bacteria in PBS at the appropriate optical density (OD) were incubated with CTV for 20 min at 37°C with rotation. The bacteria were then washed and resuspended in PBS prior to infection of cells.

### Animals.

Groups of wild-type C57BL/6 mice (6 to 8 weeks of age) were housed under specific-pathogen-free (SPF) conditions at the Trinity College Dublin Comparative Medicines facility. All animal experiments were conducted in accordance with the recommendations and guidelines of the Health Products Regulatory Authority (HPRA), the competent authority in Ireland, and in accordance with protocols approved by the Trinity College Animal Research Ethics Committee.

### Cell culture.

Bone marrow-derived dendritic cells (BMDCs) were prepared by culturing bone marrow cells isolated from C57BL/6 mice with granulocyte-macrophage colony-stimulating factor (GM-CSF), as described previously (29). On day 10, loosely adherent cells were collected, washed, reseeded at a concentration of 2 × 10^5^ cells/well in medium without antibiotics, and rested overnight.

Peritoneal macrophages were isolated as previously described (30) and seeded at 2 × 10^5^ cells/well in medium containing no antibiotics.

Immortalized bone marrow-derived macrophages (iBMMs) stably expressing enhanced GFP-LC3 (GFP-LC3) (31) were cultured in complete RPMI (cRPMI) medium under constant selection with 10 μg/ml puromycin. Cells were seeded at 1 × 10^6^ cells/well on poly-l-lysine-coated 19-mm coverslips in 12-well plates.

### Infection of phagocytes.

Cells were infected with live S. aureus bacteria at a multiplicity of infection (MOI) of 10 or 100 for the indicated times. In some cases, prior to infection, cells were incubated with 10 mM 3-methyladenine (3-MA; Sigma) for 30 min. At 2 h postinfection, medium was replaced with fresh medium containing gentamicin (200 μg/ml) for 1 h to kill extracellular bacteria. This medium was replaced with fresh medium containing no antibiotics, and this was considered time zero (*T*_0_).

For assessment of total killing, cells were infected with live S. aureus bacteria at an MOI of 10 or 100 for the indicated times and were not treated with gentamicin.

### Assessment of bacterial killing.

At the indicated time points, infected cells were spun down, the supernatant was removed, and cells were lysed by the addition of 20 μl 0.1% Triton X-100. The supernatant was then reintroduced into the well and mixed with the cell lysate. Serial dilutions of the suspension were prepared in PBS and plated onto tryptic soy agar (TSA) to determine the number of CFU/milliliter. Bacterial killing was determined as the percent reduction in the number of CFU in wells containing bacteria and phagocytes compared to the number of CFU in wells containing bacteria only.

### Assessment of bacterial escape.

S. aureus-infected BMDCs underwent gentamicin treatment as described above. At specific time points, the cell-free supernatants were collected, serially diluted in PBS, and plated onto TSA to determine the number of bacteria that had escaped into the medium, measured as the fold increase in log CFU/well from time zero.

### Cell viability assays.

To assess S. aureus-induced cytotoxicity, BMDCs were infected and treated with gentamicin as described above. Lactate dehydrogenase (LDH) release was measured by using the Pierce LDH cytotoxicity assay kit (Thermo Scientific) according to the manufacturer's instructions. In some cases, cell viability was assessed by the addition of propidium iodine (PI) (1 μg/ml; eBioscience) and analysis by flow cytometry.

### Vesicle lysis test.

Phospholipid vesicles were prepared as described previously (32). A vesicle lysis test (VLT) was performed by using a 1:1 ratio of the bacterial supernatant (cultures grown for 18 h) and pure vesicles, and fluorescence intensity was measured at excitation and emission wavelengths of 485 and 520 nm, respectively, on a FLUOstar fluorometer (BMG Labtech). Positive and negative controls were pure vesicles with 0.01% Triton X-100 and HEPES buffer, respectively.

### Measurement of RNAIII expression by reverse transcription-quantitative PCR.

S. aureus RNA was isolated by using the RNeasy minikit (Qiagen) according to the manufacturer's instructions, with the addition of Turbo DNase (Ambion) after the purification step. RNA was quantified by using the RNA broad-range kit (Qubit), and reverse transcription (RT) was performed by using the ProtoScript *Taq* RT-PCR kit (New England BioLabs) according to the manufacturer's instructions, using random primers. Standard curves were generated for both gyrase B (*gyr*FW [5′-CCAGGTAAATTAGCCGATTGC-3′] and *gyr*RV [5′-AAATCGCCTGCGTTCTAGAG]) and RNAIII (*rna*IIIFW [5′-GAAGGAGTGATTTCAATGGCACAAG-3′] and *rna*IIIRV [5′-GAAAGTAATTAATTATTCATCTTATTTTTTAGTGAATTTG-3′]) primers, using genomic DNA to determine primer efficiency. Real-time PCR was performed by using SYBR green PCR master mix (Applied Biosystems) as previously described (32).

### Western immunoblotting.

To detect LC3, BMDCs were infected and treated with gentamicin as described above. At the specified time points, BMDCs were lysed in NP-40 lysis buffer. The protein concentration of the lysates was measured by using a Bradford assay (Thermo Scientific), and equal concentrations of protein were loaded into each lane of the gel. Samples were separated on a 15% SDS-polyacrylamide gel and transferred onto a polyvinylidene difluoride (PVDF) membrane. The membrane was blocked with 5% (wt/vol) milk before being probed with antibody (rabbit anti-LC3 at 1:1,000 [Cell Signaling] and horseradish peroxidase [HRP]-conjugated goat anti-rabbit immunoglobulin G [IgG] at 1:10,000 [Jackson Immune]). The membrane was developed with enhanced chemiluminescence (ECL) (Mybio) on a Bio-Rad GelDoc system.

To detect Hla expression, proteins from the filtered bacterial supernatant were concentrated by trichloroacetic acid precipitation, separated on a 12.5% SDS-polyacrylamide gel, and transferred onto a PVDF membrane. The membrane was blocked in 10% (wt/vol) milk and probed with polyclonal rabbit anti-Hla IgG (1:1,000) (33), followed by HRP-conjugated protein A (Sigma). Reactive bands were visualized by using the LumiGLO reagent and peroxide detection system (Cell Signaling Technology).

### Confocal imaging.

BMDCs were infected and treated with gentamicin as described above, and monodansylcadaverine (MDC) (50 μM) was added 15 min prior to cell fixation. Cells were then fixed in 2% paraformaldehyde (PFA; Thermo Scientific). Alternatively, GFP-positive (GFP^+^) LC3 BMMs were infected and treated with gentamicin as described above. At specific time points postinfection, cells were fixed in 2% PFA and permeabilized in Triton X-100 (0.1% in PBS). Nonspecific binding was blocked by incubation in 5% bovine serum albumin (BSA) before cells were incubated with Alexa Fluor 555-conjugated phalloidin (1:100; Life Technologies) for 1 h to stain actin.

The coverslips were mounted onto glass slides with fluorescent mounting medium (DakoCytomation) and analyzed with an Olympus FV1000 confocal laser scanning microscope.

### *In vivo* intraperitoneal infection model.

Mice were infected with S. aureus (5 × 10^8^ CFU) via intraperitoneal (i.p.) injection. At specific time points postinfection, peritoneal exudate cells (PECs) were isolated by lavage of the peritoneal cavity with sterile PBS. Lavage fluid was serially diluted in PBS and plated onto TSA to determine the bacterial burden at the site of infection. Spleens were isolated and homogenized in 2 ml of sterile PBS. Tissue homogenates were then serially diluted in PBS and plated onto TSA to determine the tissue bacterial burden. Blood was collected by cardiac puncture with a 27-gauge needle and a heparinized 1-ml syringe. The CFU/milliliter of blood was determined by serial dilution and plating onto TSA plates.

To isolate leukocytes, blood was layered onto Histopaque-1083 (Sigma) for density gradient centrifugation. Leukocytes were collected between the plasma layer and the pellet containing red blood cells (RBCs) and extracellular bacteria (34). Isolated leukocytes were then washed well and resuspended in Fcγ block for flow cytometric analysis or lysed in sterile water to quantify cell-associated CFU.

### Flow cytometry.

PECs or blood leukocytes were blocked in Fcγ block (1 μg/ml; eBioscience) and then surface stained with fluorochrome-conjugated antibodies against Ly6G (clone 1A8; BD Bioscience), F4/80 (clone BM8; eBioscience), CD11c (clone N418; eBioscience), and CD11b (clone M1/70; eBioscience). Flow cytometric data were acquired with a BD FACSCanto II instrument (BD Biosciences) and analyzed using FlowJo software (Tree Star).

To assess the rate of S. aureus phagocytosis by BMDCs, cells that had been infected with CTV-labeled S. aureus for 30 min or 2 h were incubated with gentamicin (200 μg/ml) for 1 h, washed, and fixed in 2% PFA. The cells were then analyzed on BD FACSCanto II by gating on forward scatter and side scatter, and the percentage of CTV^+^ cells was assessed.

### Statistical analysis.

Statistical analysis was carried out using GraphPad Prism statistical analysis software. Differences between groups were analyzed by the unpaired Student *t* test or analysis of variance (ANOVA) with the appropriate posttest and by using repeated measures where required. A *P* value of <0.05 was considered statistically significant.

## RESULTS

### Killing of S. aureus by dendritic cells and macrophages is strain dependent.

**FIG 1:**
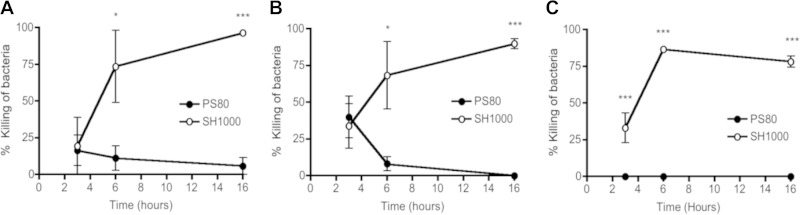
Killing of S. aureus by dendritic cells and macrophages is strain dependent. (A and B) BMDCs were infected with PS80 or SH1000 at an MOI of 10 (A) or 100 (B). (C) Alternatively, peritoneal macrophages were infected with either strain at an MOI of 100. The percent killing of bacteria was determined by comparing the number of total CFU in the presence of phagocytes to the number of CFU in medium only. Results are expressed as means ± standard errors of the means for each time point (*n* = 3 to 4). *, *P* < 0.05; ***, *P* < 0.001 (compared to other strains, as determined by repeated-measures two-way ANOVA with a Bonferroni posttest).

Despite the fact that dendritic cells have been shown to be involved in coordinating the immune response to S. aureus infection, their contribution to direct bacterial killing remains to be fully established (35, 36). We compared the bactericidal capacities of these phagocytic cells to those of macrophages, which have a more clearly defined role in direct killing of S. aureus (37). Primary BMDCs were infected with two laboratory strains of S. aureus at an MOI of 10 (Fig. 1A) and an MOI of 100 (Fig. 1B), and bacterial killing was monitored over time. Within 6 h of infection, ∼70% of SH1000 cells were killed, and by 16 h, almost 100% of SH1000 cells had been killed by the BMDCs at either MOI. In contrast, the BMDCs were unable to kill S. aureus strain PS80.

Interestingly, the ability of BMDCs to kill SH1000 appeared to be MOI dependent. It was reported previously that BMDCs were unable to kill SH1000 at an MOI of 0.1 (35). We also failed to detect any killing of SH1000 cells by BMDCs at this low MOI, but the ability of BMDCs to kill SH1000 cells by 16 h became apparent at MOIs of as low as 2 (97.7% ± 1.7% killing).

To establish if the inability to kill S. aureus strain PS80 was specific to dendritic cells, we infected primary peritoneal macrophages with both strains of S. aureus at an MOI of 100. Similar to the killing observed with BMDCs, peritoneal macrophages efficiently killed SH1000 but were unable to kill PS80 (Fig. 1C). Interestingly, in our hands, BMDCs and macrophages demonstrated similar capacities to kill S. aureus strain SH1000 (the percent killing at 16 h was 90% ± 6.8% in BMDCs, compared to 78.3% ± 6.6% in macrophages). Taken together, these results suggest that BMDCs are capable of killing S. aureus but that strain-dependent differences may impact the ability of both macrophages and BMDCs to kill the bacterium.

### S. aureus strain PS80 but not strain SH1000 can escape from dendritic cells, causing associated cytotoxicity.

**FIG 2:**
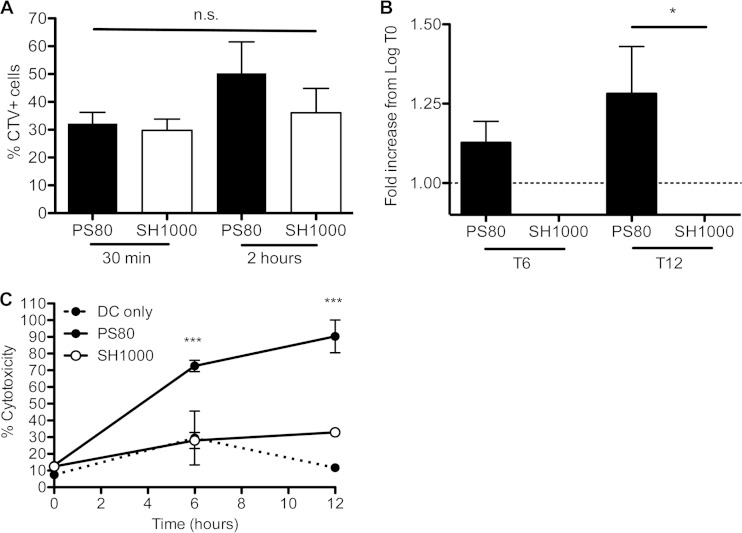
S. aureus strain PS80 but not SH1000 can escape from dendritic cells, causing associated cytotoxicity. (A) BMDCs were infected with either CTV-labeled PS80 or SH1000 at an MOI of 100. The percent uptake of bacteria was measured at 30 min or 2 h. (B) Following infection of BMDCs with PS80 or SH1000 at an MOI of 100, the escape of each strain into the cell culture medium was assessed at 6 h and 12 h. (C) LDH levels in the supernatants of both infected and uninfected BMDCs were assessed. Results are expressed as means ± standard errors of the means (A and B) or means ± standard deviations (C) (*n* = 3 to 4 [A and B]; data in panel C are representative of data from 3 independent experiments). *, *P* < 0.05; ***, *P* < 0.001; n.s., not significant (as determined by repeated-measures one- or two-way ANOVA with the appropriate posttest).

Given that BMDCs had different capacities to kill S. aureus strains PS80 and SH1000, we wanted to confirm that both strains were phagocytosed by BMDCs at the same rate. BMDCs were infected with CTV-labeled S. aureus at an MOI of 100, and the uptake of bacteria into BMDCs was assessed after 30 min and 2 h, following gentamicin treatment to kill any bacteria that had not been phagocytosed. At 30 min postinfection, PS80 and SH1000 were phagocytosed by BMDCs to the same extent, with ∼30% of BMDCs staining positively for CTV-labeled PS80 or SH1000 (Fig. 2A). At 2 h postinfection, the percentage of cells that were PS80-CTV^+^ increased, alluding to the survival of this strain inside the cells.

S. aureus strains SH1000 and PS80 were both phagocytosed by BMDCs to the same extent, but following phagocytosis, PS80 was not killed. To establish whether PS80 escaped from the BMDCs, cells were allowed to phagocytose the bacteria, and any extracellular bacteria were killed by the addition of the bactericidal antibiotic gentamicin. Cells were washed and incubated in fresh medium, and the escape of viable bacteria into the supernatant was measured after 6 and 12 h of incubation. By 6 h, there was evidence of PS80 but not SH1000 escaping from the BMDCs. By 12 h, the level of PS80 in the cell culture supernatant was significantly higher than that of SH1000 (Fig. 2B). Similar results were obtained following infection at an MOI of 10 (data not shown).

To establish if the escape of S. aureus from BMDCs was associated with cytotoxicity, LDH release from the infected BMDCs was measured. LDH activities were similar in uninfected BMDCs and BMDCs infected with SH1000 at both 6 h and 12 h postinfection, indicating that SH1000 had no effect on the viability of the infected cells. In contrast, BMDCs infected with PS80 had significantly higher levels of LDH in the supernatant than did BMDCs infected with SH1000 or uninfected BMDCs at both time points (Fig. 2C), indicating significant cytotoxicity.

### Identification of S. aureus bloodstream infection isolates with the ability to escape phagocytic killing.

**FIG 3:**
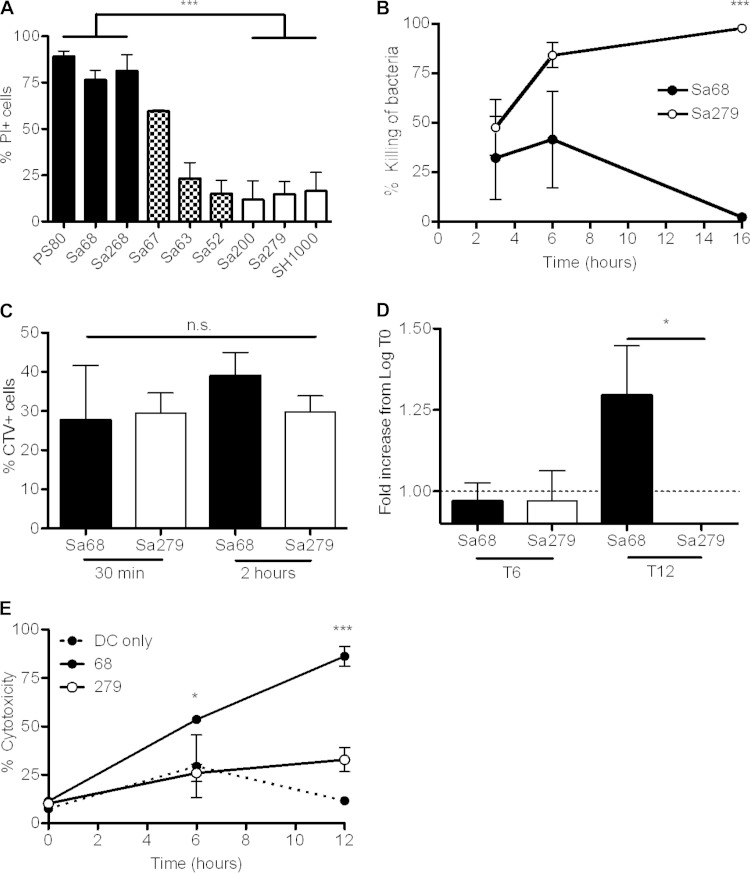
Identification of clinical bloodstream isolates with the ability to escape phagocytic killing. (A) BMDC viability was screened by PI staining at 24 h postinfection with a panel of clinical strains, identifying PS80-like strains (black bars), SH1000-like strains (white bars), and “intermediate” strains (checkered bars). (B) BMDCs were infected with Sa68 or Sa279, and the percent killing of bacteria was determined by comparing the number of total CFU in the presence of phagocytes to the number of CFU in medium only. (C) BMDCs were infected with either CTV-labeled Sa68 or Sa279 at an MOI of 100, and the percent uptake of each strain was determined by flow cytometry at 30 min or 2 h postinfection. (D) Following infection of BMDCs with Sa68 or Sa279 at an MOI of 100, the escape of each strain into the cell culture medium was assessed at 6 h and 12 h. (E) LDH levels in the supernatants of both infected and uninfected BMDCs were assessed. Results are expressed as means ± standard errors of the means (A to D) or means ± standard deviations (E) (*n* = 2 to 6 [A to D]; data in panel E are representative of data from 3 independent experiments). *, *P* < 0.05; ***, *P* < 0.001 (as determined by repeated-measures one- or two-way ANOVA with the appropriate posttest).

S. aureus PS80 and SH1000 are both well-characterized laboratory strains. However, their relevance to clinical isolates may be limited. Accordingly, isolates that were recovered from S. aureus bacteremia patients were collected and screened for cytotoxic effects. BMDCs were infected with each isolate at an MOI of 100, and the viability of the infected DCs was assessed after 24 h by staining with PI. The clinical isolates clustered together into one group that was cytotoxic to BMDCs, similarly to PS80; a second group that did not exert any cytotoxic effects, akin to SH1000; and a third, intermediate group (Fig. 3A).

Representative isolates from both the “PS80-like” group and the “SH1000-like” group were selected for analysis, S. aureus 68 (Sa68) and S. aureus 279 (Sa279). BMDCs were infected with Sa68 or Sa279 at an MOI of 100. The BMDCs were capable of killing strain Sa279 but were unable to kill strain Sa68 (Fig. 3B). These data suggest that Sa68 is similar to PS80 and may be capable of escaping from phagocytes. We confirmed that both strains were phagocytosed by BMDCs at similar rates by labeling the bacteria with CTV and infecting BMDCs as described above. Similar to the uptake of PS80 and SH1000, ∼30% of BMDCs were associated with CTV^+^ Sa279 or Sa68 by 30 min postinfection (Fig. 3C). We then assessed the ability of Sa68 to escape from BMDCs. After 12 h, the level of Sa68 in the cell culture supernatant was significantly higher than that of Sa279 (Fig. 3D).

To establish if the ability of Sa68 to escape from BMDCs correlated with cytotoxicity, cells were infected with Sa68 or Sa279 or were left uninfected. Following gentamicin killing of extracellular nonphagocytosed bacteria, LDH release was monitored at 6 h and 12 h. The level of cytotoxicity (LDH release) associated with Sa68-infected cells was significantly higher than that associated with Sa279-infected cells or uninfected control cells (Fig. 3E).

### Infection with PS80, but not SH1000, is associated with increased accumulation of LC3-II^+^ autophagosomes.

**FIG 4:**
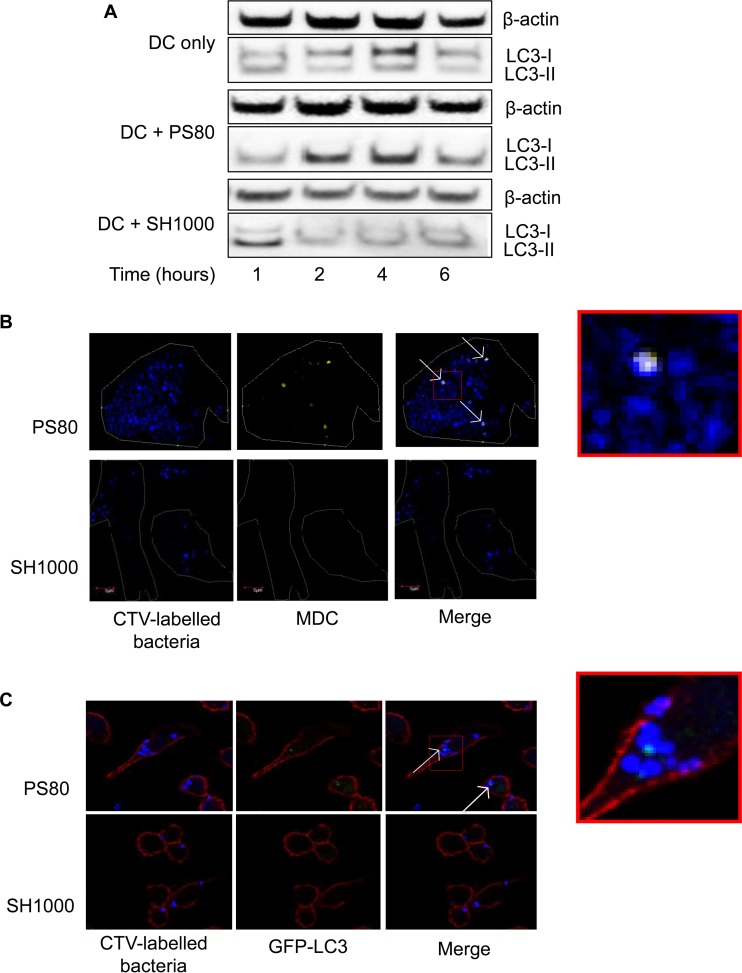
S. aureus strain PS80 inhibits normal autophagic flux in phagocytes. (A) BMDCs were infected with S. aureus strain PS80 or SH1000. At the indicated time points, cells were lysed, and expression of LC3 was analyzed by Western immunoblotting. Bands show the conversion of LC3-I to LC3-II. The level of β-actin was measured as a loading control. Representative blots from 3 independent experiments are shown. (B) Six hours after infection with CTV-labeled bacteria, BMDCs were stained with MDC and fixed to be viewed under a fluorescence microscope. Blue, bacteria; yellow, MDC. White arrows indicate colocalization of bacteria and LC3-II. (C) Three hours after infection with CTV-labeled bacteria, GFP-LC3 iBMMs were fixed, permeabilized, and stained with phalloidin for viewing under a fluorescence microscope. Blue, bacteria; green, LC3; red, phalloidin. White arrows indicate colocalization of bacteria and LC3-II. See also the enlarged images, which show the extent of colocalization.

S. aureus was previously shown to associate with autophagosomes in nonprofessional phagocytic cells. This provides a niche for the intracellular survival of S. aureus, where it can replicate and eventually escape into the cytoplasm, ultimately leading to host cell death (23, 38). We postulated that S. aureus strain PS80 might employ a similar mechanism in BMDCs to evade killing. To assess autophagy in BMDCs, cells were infected, and lysates were prepared at intervals up to 6 h postinfection, with gentamicin killing of extracellular bacteria. Processing of the autophagic marker LC3 was then assessed by Western immunoblotting (39). Infection of BMDCs with S. aureus strain PS80 resulted in the persistence of substantial levels of LC3-II for at least 6 h. In comparison, uninfected BMDCs or BMDCs infected with SH1000 showed no accumulation of LC3^+^ autophagosomes, although there was persistently a low level of LC3-II processing, which was presumably due to homeostatic autophagy, followed by autosome-lysosome fusion and degradation of LC3 (Fig. 4A).

To confirm that PS80 was associating with autophagosomes, BMDCs were infected with CTV-labeled S. aureus strain PS80 or SH1000 and then treated with gentamicin to kill any extracellular bacteria. Staining with MDC (a fluorescent compound which accumulates specifically in autophagic vacuoles [40]) revealed colocalization between PS80 and the autophagosome. SH1000 was not seen to colocalize to the same extent (Fig. 4B). Additionally, RAW 264 macrophages that had been stably transfected with GFP-LC3 (31) were infected and treated with gentamicin as described above. Again, CTV-labeled PS80 was seen to colocalize with GFP-LC3 punctae at 3 h postinfection. In comparison, SH1000 did not show the same level of association with GFP-LC3 punctae (Fig. 4C).

**FIG 5:**
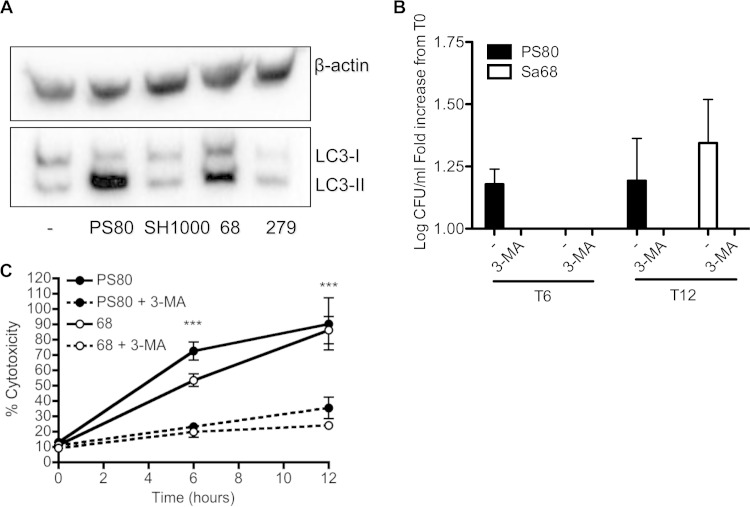
Inhibition of autophagic flux facilitates escape of S. aureus from phagocytes. (A) BMDCs were infected with S. aureus strains PS80, SH1000, Sa68, and Sa279. At 6 h, cells were lysed, and expression of LC3 was analyzed by Western immunoblotting. Bands show the conversion of LC3-I to LC3-II. The level of β-actin was measured as a loading control. A representative blot is shown. (B) BMDCs were pretreated with 3-MA for 30 min and infected with either PS80 or Sa68 (MOI of 100). The escape of each strain into the cell culture medium was assessed at 6 h and 12 h. (C) LDH levels in the supernatants of 3-MA-pretreated and untreated BMDCs that were infected with either PS80 or Sa68 were assessed. Results are expressed as means ± standard errors of the means (A and B) or means ± standard deviations (C) (*n* = 4 to 6 [A and B]; data in panel C are representative of data from 3 independent experiments). ***, *P* < 0.001 (as determined by repeated-measures two-way ANOVA with a Bonferroni posttest).

To confirm that clinical isolates could also manipulate the autophagic process, BMDCs were infected with Sa68 or Sa279, and lysates were prepared after 6 h. Processing of LC3 was assessed by Western immunoblotting. Similarly to PS80, Sa68-infected cells had considerable levels of LC3-II present, indicating a delay in the degradation of the autophagosomes. In addition, the level of LC3-II in Sa279-infected cells was similar to that in SH1000-infected cells or uninfected BMDCs, suggesting that these cells had normal autophagic flux (Fig. 5A).

### Engagement of autophagosomes facilitates escape of S. aureus from phagocytes.

To ascertain if the delay in the turnover of autophagosomes was associated with the ability of S. aureus strains PS80 and Sa68 to escape phagocyte killing, BMDCs were pretreated with 3-methyladenine (3-MA), a well-established phosphatidylinositol 3-kinase (PI3K) inhibitor that inhibits the induction of autophagy (42), prior to infection with these two strains. The escape of S. aureus into the supernatant was then assessed at 6 and 12 h. In the presence of 3-MA, the escape of PS80 and Sa68 into the cell culture supernatant was completely inhibited (Fig. 5B). Associated with this, 3-MA treatment restored the viability of the infected BMDCs, with the level of LDH activity in the culture supernatant being significantly reduced following infection with both PS80 and Sa68 (Fig. 5C). Importantly, 3-MA had no direct effect on bacterial viability after 18 h of incubation (7.90 ± 0.13 versus 7.55 ± 0.39 log CFU/ml for S. aureus alone versus S. aureus plus 3-MA).

### Differential expression of Agr by S. aureus strains correlates with their ability to engage autophagosomes.

**FIG 6:**
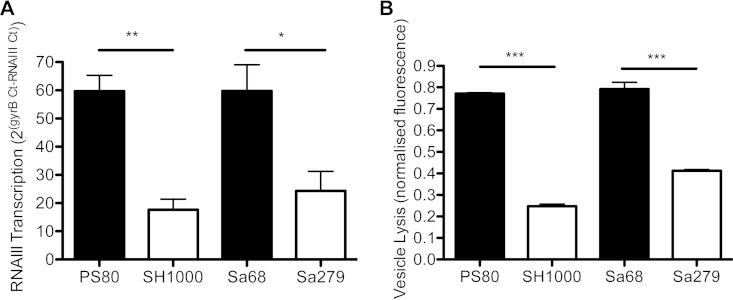
S. aureus strains exhibit distinct levels of Agr activity, as assessed by VLT and RNAIII gene expression. (A) The bacterial supernatant was incubated with lipid vesicles at a 1:1 ratio, and the fluorescence intensity was recorded as a measure of vesicle lysis. (B) RNAIII activity was measured by using quantitative RT-PCR, as a ratio of RNAIII to *gyrB* transcript numbers. Results are expressed as means ± standard errors of the means (*n* = 3 to 4). *, *P* < 0.05; **, *P* < 0.01; ***, *P* < 0.001 (as determined by one-way ANOVA with a Tukey posttest).

It was previously shown that the ability of S. aureus to divert from the endosomal pathway to autophagosomes is driven by factors that are under the control of the Agr regulatory system (23). We hypothesized that the different abilities of strains to delay autophagic flux may be associated with the level of expression of Agr. Consequently, Agr activity was measured by using a vesicle lysis test (VLT). This assay measures the interaction of PSM toxins with lipid vesicles (32). The PSMα peptide delta-toxin is translated from a short open reading frame located within the regulatory RNAIII molecule, while transcription of the other *psm* genes is activated directly by the AgrA response regulator of the Agr two-component signal transduction system that responds to high cell density. Expression of these membrane-damaging toxins is a direct manifestation of the level of expression of Agr in the stationary phase of growth (43). S. aureus strains PS80 and Sa68 induced significantly more vesicle lysis than did SH1000 and Sa279 (Fig. 6A), suggesting a higher level of Agr activity in these strains. To further assess the expression of Agr, the RNAIII level was measured. Consistent with data from the VLT, RNAIII was expressed at higher levels by S. aureus strains PS80 and Sa68 than by SH1000 and Sa279 (Fig. 6B). Taken together, we can conclude that S. aureus strains PS80 and Sa68, which induce autophagosome accumulation, exhibit a higher level of Agr activity than do SH1000 and Sa279, which have no effect on autophagosomes.

### Deletion of the *agr* locus prevents LC3-II accumulation and facilitates bacterial killing.

**FIG 7:**
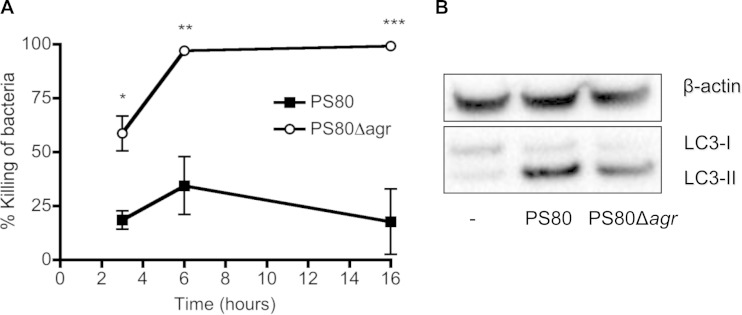
PS80Δ*agr* is killed by BMDCs and leads to reduced accumulation of LC3-II. (A) BMDCs were infected with S. aureus strain PS80 or PS80Δ*agr* at an MOI of 100. The percent killing of bacteria was determined by comparing the number of total CFU in the presence of BMDCs to the number of CFU in medium only. (B) At 6 h, cells were lysed, and expression of LC3 was analyzed by Western immunoblotting. The level of β-actin was measured as a loading control. Results are expressed as means ± standard errors of the means (*n* = 3 [A]; data in panel B are representative of data from 3 individual experiments). *, *P* < 0.05; **, *P* < 0.01; ***, *P* < 0.001 (as determined by repeated-measures two-way ANOVA with a Bonferroni posttest).

In order to investigate if strain-dependent differences in bacterial killing and the delay of normal autophagic flux were under the control of Agr-regulated genes, we generated an *agr* mutant strain of PS80 by allelic exchange. BMDCs were infected with PS80 and PS80Δ*agr*, and bacterial killing was monitored over time. By 6 h postinfection, almost 100% of PS80Δ*agr* bacteria were killed (Fig. 7A), compared to the parental strain, which failed to be killed. Furthermore, the escape of PS80 from BMDCs was significantly inhibited in the absence of *agr* (1.29 ± 0.28-fold increase in log CFU/well compared to PS80 at *T*_0_ versus 0.52 ± 0.12-fold reduction in log CFU/well compared to PS80Δ*agr* at *T*_0_) at 12 h postinfection.

In addition, the accumulation of LC3-II in infected BMDCs was also measured after 6 h of infection with PS80 or PS80Δ*agr*. LC3-II expression was reduced in cells infected with PS80Δ*agr* compared to the wild type, further proving that the *agr* locus plays a role in the ability of PS80 to block autophagic flux. However, LC3-II processing was not reduced to baseline levels (Fig. 7B), suggesting that PS80 may be expressing alternative, non-Agr-regulated genes, which have some capacity to delay autophagic flux.

### Agr influences S. aureus persistence *in vivo*.

Having established that both laboratory and clinical strains of S. aureus can subvert autophagy to evade phagocytic killing, it was important to determine whether this phenomenon affected infection outcomes *in vivo*. Groups of wild-type mice were infected with S. aureus strain PS80, SH1000, Sa68, Sa279, or PS80Δ*agr* by i.p. injection. At 3 h postchallenge, blood was collected, and the total bacterial burden in the blood was quantified (Table 2). As expected, there were significant differences in the bacterial burdens in the blood following infection with different strains. It was previously documented that strains of S. aureus expressing capsular polysaccharide (CP) seed the bloodstream from the peritoneal cavity in larger numbers than do acapsular strains (44, 45). PS80 is known to express CP8 (24), SH1000 and PS80Δ*agr* are acapsular (25, 46), and the CP expression of the clinical strains is unknown. In order to prove that the differential abilities of these strains to seed the blood were not simply due to differences in CP expression levels, mice were infected with PS80 or an isogenic mutant of PS80, RMS-1, that is acapsular (45). At 3 h postinfection, blood was isolated, and the total bacterial burden was quantified. There was no significant difference in the levels of bacteria recoverable from the blood between the two groups (4.0 ± 0.2 versus 3.7 ± 0.1 log CFU/ml fo r PS80 versus RMS-1), confirming that the observed differences in bacteremia levels were not a result of differential CP expression.

**TABLE 2:** Bacterial burden in blood

Strain	Mean log CFU/ml ± SEM	*P* value compared to PS80^*a*^
PS80	3.63 ± 0.12	NS
SH1000	2.93 ± 0.26	<0.01**
Sa68	3.92 ± 0.39	NS
Sa279	2.37 ± 0.49	<0.001***
PS80Δ*agr*	2.28 ± 0.29	<0.0001***

a NS, not significant.

**FIG 8:**
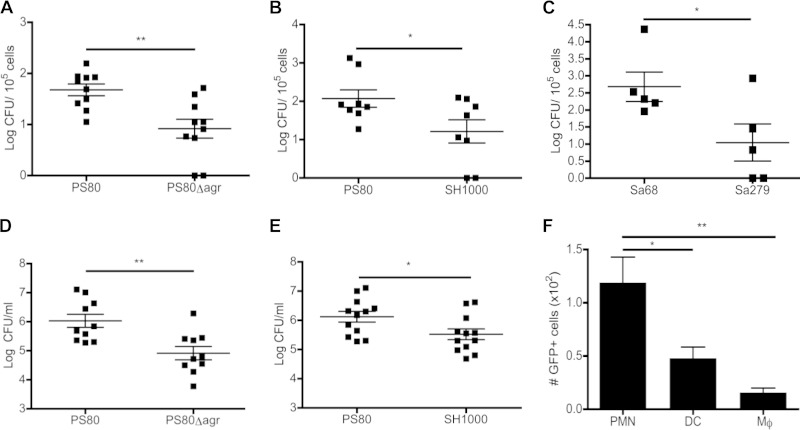
Intracellular persistence of S. aureus facilitates infection *in vivo*. Groups of mice were challenged with S. aureus strain PS80 (A, B, D, and E), PS80Δ*agr* (A and D), SH1000 (B and E), Sa68 (C), Sa279 (C), or GFP-PS80 (F) (5 × 10^8^ CFU) via the intraperitoneal route. (A to C) At 3 h postchallenge, blood was collected, and total leukocytes were isolated, washed, and lysed. Numbers of cell-associated bacteria per 10^5^ cells are shown. (D and E) At 12 h postchallenge, spleens were isolated and homogenized, and the bacterial burden was assessed. (F) Leukocytes isolated at 3 h postchallenge were also analyzed by flow cytometry, and CD11b^+^ F480^−^ Ly6G^+^ (neutrophils [PMNs]), CD11c^+^ (DCs), and CD11b^+^ F480^+^ Ly6G^−^ (monocytes [Mϕ]) populations that were GFP^+^ were determined. Results expressed as means ± standard errors of the means; the lines indicate the means (*n* = 5 to 12). *, *P* < 0.05; **, *P* < 0.01 (as determined by an unpaired Student *t* test or one-way ANOVA with a Tukey posttest).

To prove that differences in bacterial burden in the blood were due to the differential abilities of individual strains to survive intracellularly, mice were infected with S. aureus strain PS80, SH1000, PS80Δ*agr*, Sa68, or Sa279. At 3 h postinfection, the total leukocytes were separated from the RBCs and extracellular bacteria by centrifugation on Histopaque-1083. Leukocytes were then washed thoroughly and lysed to quantify viable intracellular S. aureus bacteria. The number of intracellular bacteria recovered was significantly higher in PS80-infected animals than in PS80Δ*agr*-infected (Fig. 8A) or SH1000-infected (Fig. 8B) animals. The same trend was seen for the clinical strains, with significantly higher levels of Sa68 being recovered from the blood leukocytes than Sa279 (Fig. 8C). This suggests that PS80 and Sa68 are capable of surviving within phagocytes *in vivo*, potentially facilitating systemic dissemination and persistence. Consistent with this, animals infected with PS80 demonstrated a significantly increased bacterial burden in the spleen at 12 h postchallenge compared to animals infected with PS80Δ*agr* (Fig. 8D) or SH1000 (Fig. 8E). Unfortunately, due to limitations in cell numbers, we were unable to analyze autophagic flux in individual blood leukocyte populations *ex vivo*.

Finally, to establish which specific leukocyte populations in the blood were harboring intracellular S. aureus, GFP-expressing PS80 was injected into the peritoneum. At 3 h postinfection, total leukocytes were isolated from the blood. These leukocytes were stained with a panel of antibodies against various surface markers in order to identify the phagocyte populations containing intracellular bacteria. As expected, the predominant cell type associated with GFP-expressing PS80 was found to be polymorphonuclear leukocytes (PMNs). Surprisingly, DCs accounted for the cell type that contained the second largest population of PS80-GFP^+^ cells. In contrast, only a low number of monocytes was associated with PS80-GFP^+^ (Fig. 8F). This supports the contention of this study that DCs play an important direct role in the phagocytosis and clearance of S. aureus.

## DISCUSSION

Undoubtedly, the success of S. aureus as a pathogen can be attributed to its inherent ability to disarm the host's protective immune responses. In particular, S. aureus possesses a unique arsenal of virulence factors that can circumvent the bactericidal effects of phagocytes and can manipulate these cells, even parasitizing them to facilitate an intracellular lifestyle. Here we provide significant new insights into the molecular mechanisms involved. Analysis of several S. aureus strains revealed that despite being phagocytosed to similar extents, some strains could elude phagocytic killing, subsequently lysing phagocytes and escaping. The ability to evade killing was directly associated with the capacity of these strains to inhibit normal autophagic flux within the cells. We showed that the ability of S. aureus to subvert autophagic pathways and survive within phagocytes is associated with Agr activity, as strains with lower levels of Agr exhibited normal, homeostatic turnover of autophagosomes. Moreover, we established that the level of Agr expression is directly linked with the ability of S. aureus to survive intracellularly within phagocytes *in vivo*, suggesting that this phenomenon is related to the ability of S. aureus to subvert autophagy.

Previous studies have documented a protective role for dendritic cells during S. aureus infection. Depletion of dendritic cells was associated with increased mortality during S. aureus bloodstream infection (35) and impaired bacterial clearance in an S. aureus pneumonia model (36). In both cases, the beneficial effects afforded by dendritic cells were dependent upon their ability to control the inflammatory response. In this study, we demonstrated for the first time that dendritic cells also have the potential to contribute to host protection by directly killing S. aureus. The bactericidal effects of dendritic cells were found to be comparable to those of macrophages, with both cell types being effective in reducing the growth of S. aureus strain SH1000. Consequently, we chose DCs as representative phagocytes to investigate the mechanisms by which S. aureus can parasitize these cells. Of note, our findings contrasted with those of a previously reported study, which concluded that BMDCs do not play a major role in direct killing of S. aureus (35). In that study, dendritic cells were infected with S. aureus at a very low ratio (MOI of 0.1). Given that the uptake of bacteria by macrophages has been directly linked to the MOI (47), we hypothesized that bacteria must reach a critical threshold to ensure appropriate activation of the phagocytes before phagocytic killing can occur. To test this, dendritic cell killing assays were repeated by using SH1000 at an MOI of 0.1, and no killing was observed. However, the ability of BMDCs to kill SH1000 by 16 h became apparent at MOIs of as low as 2 (97.7% ± 1.7% killing).

Our previous work demonstrated that S. aureus strains SH1000 and PS80 possess distinct capacities to activate innate signaling pathways in dendritic cells, resulting in different levels of interleukin-1β (IL-1β) production (48). Accordingly, we wanted to dissect the interaction of these particular strains with dendritic cells. Interestingly, while both primary BMDCs and peritoneal macrophages were able to kill S. aureus strain SH1000, they lacked the ability to kill PS80. PS80 avoided the bactericidal effects of phagocytes and instead escaped from the cells by inducing cell death. In contrast, once phagocytosed, SH1000 did not escape from the phagocyte, and cells that ingested this strain remained viable for up to 24 h postinfection. Importantly, both strains of S. aureus were efficiently phagocytosed by dendritic cells, implying that manipulation of the phagocyte response by PS80 was exerted once it became intracellular.

S. aureus strain PS80 was previously shown to survive intracellularly within neutrophils isolated from S. aureus surgical site infections (49). We have now demonstrated that PS80 establishes its intracellular survival niche within phagocytes through subversion of the autophagic pathway. Following infection of BMDCs, PS80 prevented the constitutive degradation of autophagosomes by lysosomes, leading to the accumulation of LC3-II. In contrast, S. aureus strain SH1000 did not interfere with the homeostatic turnover of the autophagic machinery. Furthermore, BMDCs that had been treated with MDC (which accumulates in the autophagosome) postinfection showed colocalization between the autophagosome and PS80 but not SH1000. In addition, macrophages that were stably transfected with GFP-LC3 also demonstrated colocalization of PS80 with LC3-II punctae, indicating the interaction of the bacterium with autophagosomes. Upon invasion of nonprofessional phagocytes, S. aureus was shown to subvert autophagy, enabling replication within the autophagosome and subsequent lysis of the host cell (23). Consistent with this, we have demonstrated that the cytotoxic effects exerted by S. aureus strain PS80 on BMDCs are associated with the subversion of autophagy. Treatment of BMDCs with the autophagy inhibitor 3-MA protected cells from PS80-induced cytotoxicity and simultaneously prevented the escape of the bacterium from the phagocyte.

Importantly, bloodstream infection isolates with phenotypes comparable to those of PS80 and SH1000 were identified, highlighting the clinical relevance of this phagocyte evasion strategy for facilitating systemic infection. Similarly to PS80, Sa68 was not killed by BMDCs and could escape from the cells, causing associated cytotoxicity. In contrast, Sa279 behaved more like SH1000 and was killed by BMDCs. This was consistent with the observation that Sa68 induced significant LC3-II accumulation in BMDCs, while inhibition of autophagy by using 3-MA reduced the escape of Sa68 from BMDCs.

The ability of S. aureus to subvert autophagy in nonphagocytic cells is controlled by the Agr system and has been shown to specifically depend upon Agr-regulated expression of alpha-toxin (Hla) (23, 38). *In vitro*, *agr* and *hla* mutants of S. aureus fail to trigger autophagy; are delivered efficiently to the lysosome, where they are degraded; and thus cannot survive intracellularly for extended periods. However, a recently reported *in vivo* study has shown that while autophagy plays an important role in conferring protection against S. aureus lethality by mediating tolerance to the cytotoxic effects of Hla, infection with an Hla mutant strain actually caused increased bacterial burdens in wild-type mice in comparison to Atg16L1^HM^ mice (which display reduced autophagy). This indicates that Hla may actually be dispensable in the exploitation of autophagy in the context of intracellular bacterial survival (50). Interestingly, when we profiled Hla expression among our strains, it did not correlate with the abilities of these strains to inhibit autophagic flux in phagocytes. S. aureus strains PS80 and Sa68 were comparable in their abilities to manipulate autophagy in order to evade phagocytic killing; however, PS80 was a high-level Hla producer, whereas Sa68 was Hla negative. Furthermore, both SH1000 and Sa279 are killed by DCs and fail to accumulate autophagosomes, but SH1000 expresses low levels of Hla, and no expression is detectable in Sa279 (see Fig. S1 in the supplemental material). S. aureus strains PS80 and Sa68, which evade phagocytic killing through the subversion of autophagy, express higher levels of Agr RNAIII and membrane-damaging cytolytic peptide toxins than do SH1000 and Sa279, which did not have any appreciable effect on autophagy and were killed by the phagocytes. Crucially, we have also shown that Agr activity dictated the ability of S. aureus to survive within phagocytes *in vivo*. Using an *agr* mutant of PS80, we demonstrated a reduced ability of PS80Δ*agr* to survive within leukocytes isolated from peripheral blood following systemic challenge compared to that of wild-type strain PS80. In addition, SH1000 (which exhibited reduced Agr activity) also had a significantly reduced capacity to survive within peripheral blood leukocytes *in vivo*, confirming that the inability of PS80Δ*agr* to survive in the phagocytes is not an artifact of the mutation of *agr*. Similarly, clinical strain Sa279 (which also exhibits reduced Agr activity) shows significantly reduced survival in circulating leukocytes in comparison to Sa68. It appears that the Agr-dependent predilection of PS80 and Sa68 for associating with autophagosomes enables them to survive within circulating leukocytes, thus potentially increasing their capacity for systemic dissemination. Consistent with this, bacterial burdens in the spleen were significantly elevated in PS80-infected mice compared to those in the spleen of animals infected with PS80Δ*agr* or SH1000, suggesting that intracellular survival in the autophagosome facilitates increased persistence in the periphery of the host.

Until this study, Hla was the only known S. aureus virulence factor implicated in the induction of autophagy (38). However, the pattern of Hla expression among the strains used in this study was not sufficient to explain the phenotypes observed, and it raises the question of whether other Agr-regulated factors might also be capable of manipulating autophagy. Intriguingly, the VLT used to assess Agr activity measures PSM activity in culture supernatants of S. aureus, and the pattern of vesicle lysis corresponds exactly with the observed phenotypes (32). Thus, it is tempting to speculate that these toxins may also have an as-yet-undocumented role in the induction of autophagy in phagocytic cells. Interestingly, melittin, a component of bee sting venom that is an α-helical, amphipathic antimicrobial peptide, similar to delta-toxin (51), was previously shown to induce autophagic cell death in trypanosomes (52). In addition, PSMαs trigger phagosomal escape by S. aureus in the monocytic cell line THP-1s (16), allowing the bacteria to replicate in the cytoplasm and leading to cell lysis (17). Autophagy has been shown to respond to bacteria both in the cytosol and within damaged phagosomes (53), supporting the notion that certain strains of S. aureus deliberately induce autophagy by causing damage to the phagosome. By inhibiting the digestion of the autophagosomes by the lysosomes, they then survive within autophagosomes. A comprehensive analysis of the role played by PSMs in the induction of and engagement with autophagic pathways is warranted but is beyond the scope of the current study.

The precise mechanism by which S. aureus subverts autophagosomes has yet to be defined. It was previously shown that autophagosomes may form around a phagosome that has been damaged by internalized bacteria such as Salmonella enterica (54), suggesting that both strains of S. aureus may be phagocytosed normally but that PS80 may then damage the phagosome deliberately in order to secrete itself within an autophagosome. Alternatively, Gresham et al. suggested that S. aureus can be taken up unconventionally by neutrophils via macropinocytosis into “large spacious vacuoles” (5). Other studies have shown that autophagy proteins can be recruited to single-membrane vacuoles such as macropinosomes (55). This may suggest an alternative internalization route for certain strains of S. aureus. While some strains are phagocytosed and killed by phagolysosomal fusion, others may become internalized via macropinocytosis, which facilitates the subversion of autophagic pathways in order to promote their survival.

Interestingly, PS80 can survive within several different phagocytic cell types *in vivo*. Consistent with data from previous studies (5, 49), we showed that neutrophils are the main intracellular reservoir of S. aureus. However, DCs showed higher levels of viable intracellular bacteria than did monocytes, further supporting our belief that these cells are critical in regulating the outcome of S. aureus infection. The primary role of DCs is to migrate to the lymph node following antigen uptake in order to activate the adaptive immune response. Therefore, the ability to survive within these cells may be an attractive route of dissemination for S. aureus.

This study contributes to the growing literature that links the subversion of autophagosomes by S. aureus with intracellular survival (23, 38). Our data demonstrate that S. aureus strain PS80 and a comparable clinical isolate, which express high levels of Agr, prevent constitutive degradation of LC3-II^+^ autophagosomes in order to survive and escape killing by professional phagocytes. Strains that had a lower level of Agr expression did not affect the degradation of autophagosomes in BMDCs and were efficiently killed. This study implicates autophagy as a mechanism to facilitate the temporary intracellular survival of certain S. aureus strains within different phagocytes, maximizing their potential for dissemination and persistence *in vivo*.

The notion that S. aureus could parasitize neutrophils to facilitate dissemination has already been proposed (56), and our studies support the hypothesis that other phagocytes may also act as “Trojan horses” for the metastasis of S. aureus, provided that the infecting organism possesses the appropriate tools to subvert autophagosomes. Given that our findings were replicated with clinically relevant strains, it is tempting to speculate that identification of S. aureus isolates that can inhibit autophagic flux by measuring Agr activity may predict invasive disease potential.

## Supplementary Material

Supplemental material

## References

[1] Finlay BB, McFadden G 2006 Anti-immunology: evasion of the host immune system by bacterial and viral pathogens. Cell 124:767–782. doi:10.1016/j.cell.2006.01.034. 16497587

[2] Garzoni C, Kelley WL 2009 Staphylococcus aureus: new evidence for intracellular persistence. Trends Microbiol 17:59–65. doi:10.1016/j.tim.2008.11.005. 19208480

[3] Kubica M, Guzik K, Koziel J, Zarebski M, Richter W, Gajkowska B, Golda A, Maciag-Gudowska A, Brix K, Shaw L, Foster T, Potempa J 2008 A potential new pathway for Staphylococcus aureus dissemination: the silent survival of S. aureus phagocytosed by human monocyte-derived macrophages. PLoS One 3:e1409. doi:10.1371/journal.pone.0001409. 18183290 PMC2169301

[4] Voyich JM, Braughton KR, Sturdevant DE, Whitney AR, Said-Salim B, Porcella SF, Long RD, Dorward DW, Gardner DJ, Kreiswirth BN, Musser JM, DeLeo FR 2005 Insights into mechanisms used by Staphylococcus aureus to avoid destruction by human neutrophils. J Immunol 175:3907–3919. doi:10.4049/jimmunol.175.6.3907. 16148137

[5] Gresham HD, Lowrance JH, Caver TE, Wilson BS, Cheung AL, Lindberg FP 2000 Survival of Staphylococcus aureus inside neutrophils contributes to infection. J Immunol 164:3713–3722. doi:10.4049/jimmunol.164.7.3713. 10725730

[6] Koziel J, Maciag-Gudowska A, Mikolajczyk T, Bzowska M, Sturdevant DE, Whitney AR, Shaw LN, DeLeo FR, Potempa J 2009 Phagocytosis of Staphylococcus aureus by macrophages exerts cytoprotective effects manifested by the upregulation of antiapoptotic factors. PLoS One 4:e5210. doi:10.1371/journal.pone.0005210. 19381294 PMC2668171

[7] Schlesinger LS 1996 Entry of Mycobacterium tuberculosis into mononuclear phagocytes. Curr Top Microbiol Immunol 215:71–96. 8791710 10.1007/978-3-642-80166-2_4

[8] Drevets DA 1999 Dissemination of Listeria monocytogenes by infected phagocytes. Infect Immun 67:3512–3517. 10377133 10.1128/iai.67.7.3512-3517.1999 PMC116538

[9] Derby BM, Rogers DE 1961 Studies on bacteriemia. V. The effect of simultaneous leukopenia and reticuloendothelial blockade on the early blood stream clearance of staphylococci and Escherichia coli. J Exp Med 113:1053–1066. 13722011 10.1084/jem.113.6.1053 PMC2137435

[10] Bera A, Herbert S, Jakob A, Vollmer W, Götz F 2005 Why are pathogenic staphylococci so lysozyme resistant? The peptidoglycan O-acetyltransferase OatA is the major determinant for lysozyme resistance of Staphylococcus aureus. Mol Microbiol 55:778–787. 15661003 10.1111/j.1365-2958.2004.04446.x

[11] Liu GY, Essex A, Buchanan JT, Datta V, Hoffman HM, Bastian JF, Fierer J, Nizet V 2005 Staphylococcus aureus golden pigment impairs neutrophil killing and promotes virulence through its antioxidant activity. J Exp Med 202:209–215. doi:10.1084/jem.20050846. 16009720 PMC2213009

[12] Karavolos MH, Horsburgh MJ, Ingham E, Foster SJ 2003 Role and regulation of the superoxide dismutases of Staphylococcus aureus. Microbiology 149:2749–2758. doi:10.1099/mic.0.26353-0. 14523108

[13] Shompole S, Henon KT, Liou LE, Dziewanowska K, Bohach GA, Bayles KW 2003 Biphasic intracellular expression of Staphylococcus aureus virulence factors and evidence for Agr-mediated diffusion sensing. Mol Microbiol 49:919–927. doi:10.1046/j.1365-2958.2003.03618.x. 12890018

[14] Jarry TM, Memmi G, Cheung AL 2008 The expression of alpha-haemolysin is required for Staphylococcus aureus phagosomal escape after internalization in CFT-1 cells. Cell Microbiol 10:1801–1814. doi:10.1111/j.1462-5822.2008.01166.x. 18466345

[15] Giese B, Glowinski F, Paprotka K, Dittmann S, Steiner T, Sinha B, Fraunholz MJ 2011 Expression of delta-toxin by Staphylococcus aureus mediates escape from phago-endosomes of human epithelial and endothelial cells in the presence of beta-toxin. Cell Microbiol 13:316–329. doi:10.1111/j.1462-5822.2010.01538.x. 20946243

[16] Grosz M, Kolter J, Paprotka K, Winkler AC, Schafer D, Chatterjee SS, Geiger T, Wolz C, Ohlsen K, Otto M, Rudel T, Sinha B, Fraunholz M 2014 Cytoplasmic replication of Staphylococcus aureus upon phagosomal escape triggered by phenol-soluble modulin alpha. Cell Microbiol 16:451–465. doi:10.1111/cmi.12233. 24164701 PMC3969633

[17] Surewaard BG, de Haas CJ, Vervoort F, Rigby KM, DeLeo FR, Otto M, van Strijp JA, Nijland R 2013 Staphylococcal alpha-phenol soluble modulins contribute to neutrophil lysis after phagocytosis. Cell Microbiol 15:1427–1437. doi:10.1111/cmi.12130. 23470014 PMC4784422

[18] Kabeya Y, Mizushima N, Ueno T, Yamamoto A, Kirisako T, Noda T, Kominami E, Ohsumi Y, Yoshimori T 2000 LC3, a mammalian homologue of yeast Apg8p, is localized in autophagosome membranes after processing. EMBO J 19:5720–5728. doi:10.1093/emboj/19.21.5720. 11060023 PMC305793

[19] Mizushima N, Yoshimori T, Levine B 2010 Methods in mammalian autophagy research. Cell 140:313–326. doi:10.1016/j.cell.2010.01.028. 20144757 PMC2852113

[20] Nakagawa I, Amano A, Mizushima N, Yamamoto A, Yamaguchi H, Kamimoto T, Nara A, Funao J, Nakata M, Tsuda K, Hamada S, Yoshimori T 2004 Autophagy defends cells against invading group A Streptococcus. Science 306:1037–1040. doi:10.1126/science.1103966. 15528445

[21] Gutierrez MG, Master SS, Singh SB, Taylor GA, Colombo MI, Deretic V 2004 Autophagy is a defense mechanism inhibiting BCG and Mycobacterium tuberculosis survival in infected macrophages. Cell 119:753–766. doi:10.1016/j.cell.2004.11.038. 15607973

[22] Huang J, Klionsky DJ 2007 Autophagy and human disease. Cell Cycle 6:1837–1849. doi:10.4161/cc.6.15.4511. 17671424

[23] Schnaith A, Kashkar H, Leggio SA, Addicks K, Kronke M, Krut O 2007 Staphylococcus aureus subvert autophagy for induction of caspase-independent host cell death. J Biol Chem 282:2695–2706. doi:10.1074/jbc.M609784200. 17135247

[24] Tzianabos AO, Wang JY, Lee JC 2001 Structural rationale for the modulation of abscess formation by Staphylococcus aureus capsular polysaccharides. Proc Natl Acad Sci U S A 98:9365–9370. doi:10.1073/pnas.161175598. 11470905 PMC55426

[25] Horsburgh MJ, Aish JL, White IJ, Shaw L, Lithgow JK, Foster SJ 2002 SigmaB modulates virulence determinant expression and stress resistance: characterization of a functional rsbU strain derived from Staphylococcus aureus 8325-4. J Bacteriol 184:5457–5467. doi:10.1128/JB.184.19.5457-5467.2002. 12218034 PMC135357

[26] Cormack BP, Valdivia RH, Falkow S 1996 FACS-optimized mutants of the green fluorescent protein (GFP). Gene 173:33–38. doi:10.1016/0378-1119(95)00685-0. 8707053

[27] Monk IR, Shah IM, Xu M, Tan MW, Foster TJ 2012 Transforming the untransformable: application of direct transformation to manipulate genetically Staphylococcus aureus and Staphylococcus epidermidis. mBio 3(2):e00277-11. doi:10.1128/mBio.00277-11. 22434850 PMC3312211

[28] Li M, Elledge S 2012 SLIC: a method for sequence- and ligation-independent cloning, p 51–59. *In* Peccoud J (ed), Gene synthesis, vol 852 Humana Press, Totowa, NJ. 10.1007/978-1-61779-564-0_5 22328425

[29] Lutz MB, Kukutsch N, Ogilvie ALJ, Rößner S, Koch F, Romani N, Schuler G 1999 An advanced culture method for generating large quantities of highly pure dendritic cells from mouse bone marrow. J Immunol Methods 223:77–92. doi:10.1016/S0022-1759(98)00204-X. 10037236

[30] Murphy AG, O'Keeffe KM, Lalor SJ, Maher BM, Mills KHG, McLoughlin RM 2014 Staphylococcus aureus infection of mice expands a population of memory γδ T cells that are protective against subsequent infection. J Immunol 192:3697–3708. doi:10.4049/jimmunol.1303420. 24623128 PMC3979672

[31] Harris J, Hartman M, Roche C, Zeng SG, O'Shea A, Sharp FA, Lambe EM, Creagh EM, Golenbock DT, Tschopp J, Kornfeld H, Fitzgerald KA, Lavelle EC 2011 Autophagy controls IL-1beta secretion by targeting pro-IL-1beta for degradation. J Biol Chem 286:9587–9597. doi:10.1074/jbc.M110.202911. 21228274 PMC3058966

[32] Laabei M, Jamieson WD, Massey RC, Jenkins AT 2014 Staphylococcus aureus interaction with phospholipid vesicles—a new method to accurately determine accessory gene regulator (agr) activity. PLoS One 9:e87270. doi:10.1371/journal.pone.0087270. 24498061 PMC3907525

[33] Dajcs JJ, Austin MS, Sloop GD, Moreau JM, Hume EB, Thompson HW, McAleese FM, Foster TJ, O'Callaghan RJ 2002 Corneal pathogenesis of Staphylococcus aureus strain Newman. Invest Ophthalmol Vis Sci 43:1109–1115. 11923253

[34] Tan J, Lee BD, Polo-Parada L, Sengupta S 2012 Kinetically limited differential centrifugation as an inexpensive and readily available alternative to centrifugal elutriation. Biotechniques 53:104–108. doi:10.2144/0000113853. 23030063

[35] Schindler D, Gutierrez MG, Beineke A, Rauter Y, Rohde M, Foster S, Goldmann O, Medina E 2012 Dendritic cells are central coordinators of the host immune response to Staphylococcus aureus bloodstream infection. Am J Pathol 181:1327–1337. doi:10.1016/j.ajpath.2012.06.039. 22885107

[36] Martin FJ, Parker D, Harfenist BS, Soong G, Prince A 2011 Participation of CD11c(+) leukocytes in methicillin-resistant Staphylococcus aureus clearance from the lung. Infect Immun 79:1898–1904. doi:10.1128/IAI.01299-10. 21402768 PMC3088152

[37] Baughn R, Bonventre PF 1975 Phagocytosis and intracellular killing of Staphylococcus aureus by normal mouse peritoneal macrophages. Infect Immun 12:346–352. 807524 10.1128/iai.12.2.346-352.1975 PMC415290

[38] Mestre MB, Fader CM, Sola C, Colombo MI 2010 Alpha-hemolysin is required for the activation of the autophagic pathway in Staphylococcus aureus-infected cells. Autophagy 6:110–125. doi:10.4161/auto.6.1.10698. 20110774

[39] Rubinsztein DC, Cuervo AM, Ravikumar B, Sarkar S, Korolchuk V, Kaushik S, Klionsky DJ 2009 In search of an “autophagomometer.” Autophagy 5:585–589. doi:10.4161/auto.5.5.8823. 19411822

[40] Munafo DB, Colombo MI 2001 A novel assay to study autophagy: regulation of autophagosome vacuole size by amino acid deprivation. J Cell Sci 114:3619–3629. 11707514 10.1242/jcs.114.20.3619

[41] Reference deleted.

[42] Petiot A, Ogier-Denis E, Blommaart EF, Meijer AJ, Codogno P 2000 Distinct classes of phosphatidylinositol 3′-kinases are involved in signaling pathways that control macroautophagy in HT-29 cells. J Biol Chem 275:992–998. doi:10.1074/jbc.275.2.992. 10625637

[43] Otto M 2014 Phenol-soluble modulins. Int J Med Microbiol 304:164–169. doi:10.1016/j.ijmm.2013.11.019. 24447915 PMC4014003

[44] Thakker M, Park JS, Carey V, Lee JC 1998 Staphylococcus aureus serotype 5 capsular polysaccharide is antiphagocytic and enhances bacterial virulence in a murine bacteremia model. Infect Immun 66:5183–5189. 9784520 10.1128/iai.66.11.5183-5189.1998 PMC108646

[45] Watts A, Ke D, Wang Q, Pillay A, Nicholson-Weller A, Lee JC 2005 Staphylococcus aureus strains that express serotype 5 or serotype 8 capsular polysaccharides differ in virulence. Infect Immun 73:3502–3511. doi:10.1128/IAI.73.6.3502-3511.2005. 15908379 PMC1111869

[46] Luong T, Sau S, Gomez M, Lee JC, Lee CY 2002 Regulation of Staphylococcus aureus capsular polysaccharide expression by agr and sarA. Infect Immun 70:444–450. doi:10.1128/IAI.70.2.444-450.2002. 11796569 PMC127668

[47] Gog JR, Murcia A, Osterman N, Restif O, McKinley TJ, Sheppard M, Achouri S, Wei B, Mastroeni P, Wood JL, Maskell DJ, Cicuta P, Bryant CE 2012 Dynamics of Salmonella infection of macrophages at the single cell level. J R Soc Interface 9:2696–2707. doi:10.1098/rsif.2012.0163. 22552918 PMC3427505

[48] Maher BM, Mulcahy ME, Murphy AG, Wilk M, O'Keeffe KM, Geoghegan JA, Lavelle EC, McLoughlin RM 2013 Nlrp-3-driven interleukin 17 production by gammadeltaT cells controls infection outcomes during Staphylococcus aureus surgical site infection. Infect Immun 81:4478–4489. doi:10.1128/IAI.01026-13. 24082072 PMC3837970

[49] McLoughlin RM, Lee JC, Kasper DL, Tzianabos AO 2008 IFN-gamma regulated chemokine production determines the outcome of Staphylococcus aureus infection. J Immunol 181:1323–1332. doi:10.4049/jimmunol.181.2.1323. 18606687

[50] Maurer K, Reyes-Robles T, Alonzo F III, Durbin J, Torres VJ, Cadwell K 2015 Autophagy mediates tolerance to Staphylococcus aureus alpha-toxin. Cell Host Microbe 17:429–440. doi:10.1016/j.chom.2015.03.001. 25816775 PMC4392646

[51] Verdon J, Girardin N, Lacombe C, Berjeaud J-M, Héchard Y 2009 δ-Hemolysin, an update on a membrane-interacting peptide. Peptides 30:817–823. doi:10.1016/j.peptides.2008.12.017. 19150639

[52] Adade CM, Oliveira IRS, Pais JAR, Souto-Padrón T 2013 Melittin peptide kills Trypanosoma cruzi parasites by inducing different cell death pathways. Toxicon 69:227–239. doi:10.1016/j.toxicon.2013.03.011. 23562368

[53] Huang J, Brumell JH 2014 Bacteria-autophagy interplay: a battle for survival. Nat Rev Microbiol 12:101–114. doi:10.1038/nrmicro3160. 24384599 PMC7097477

[54] Birmingham CL, Smith AC, Bakowski MA, Yoshimori T, Brumell JH 2006 Autophagy controls Salmonella infection in response to damage to the Salmonella-containing vacuole. J Biol Chem 281:11374–11383. doi:10.1074/jbc.M509157200. 16495224

[55] Florey O, Kim SE, Sandoval CP, Haynes CM, Overholtzer M 2011 Autophagy machinery mediates macroendocytic processing and entotic cell death by targeting single membranes. Nat Cell Biol 13:1335–1343. doi:10.1038/ncb2363. 22002674 PMC3223412

[56] Thwaites GE, Gant V 2011 Are bloodstream leukocytes Trojan horses for the metastasis of Staphylococcus aureus? Nat Rev Microbiol 9:215–222. doi:10.1038/nrmicro2508. 21297670

