# Supplementary material for: Manipulation of Autophagy in Phagocytes Facilitates Staphylococcus aureus Bloodstream Infection
Source: Infect Immun. 2015 Aug 12;83(9):3445–57. doi: 10.1128/IAI.00358-15 (PMC4534639; doi:10.1128/IAI.00358-15)
Supplement: Supplemental material [file supp_83_9_3445__index.html]

Manipulation of Autophagy in Phagocytes Facilitates Staphylococcus aureus Bloodstream Infection — Supplemental material 

# Manipulation of Autophagy in Phagocytes Facilitates Staphylococcus aureus Bloodstream Infection

## Supplemental material

- Supplemental file 1 -

  Fig. S1. Hla expression of *S. aureus* strains.

  PDF, 218K
