# Supplementary material for: Manipulation of Autophagy in Phagocytes Facilitates Staphylococcus aureus Bloodstream Infection
Source: Infect Immun. 2015 Aug 12;83(9):3445–57. doi: 10.1128/IAI.00358-15 (PMC4534639; doi:10.1128/IAI.00358-15)
Supplement: Supplemental material [file IAI.00358-15_zii999091360so1.pdf]

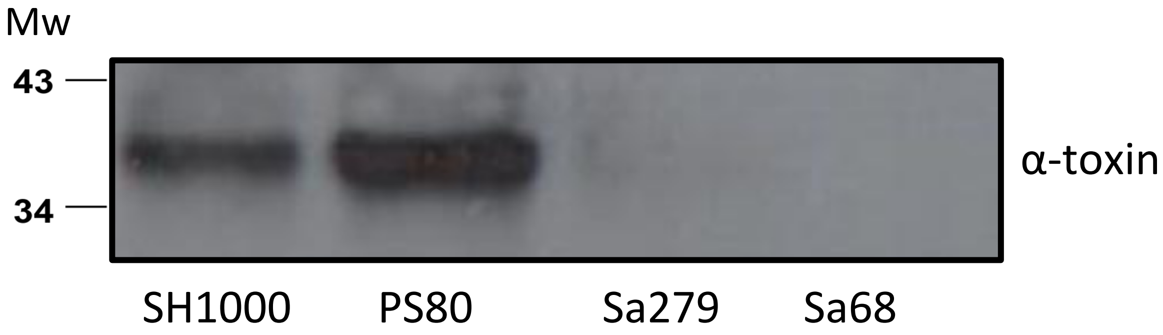

Supplemental Figure 1: Hla expression of *S. aureus* strains
Supernatants from stationary phase cultures were filtered, concentrated and expression of Hla assessed by Western blotting. A representative blot from 3 independent experiments is shown.
